# ApoE-Targeting Increases the Transfer of Solid Lipid Nanoparticles with Donepezil Cargo across a Culture Model of the Blood–Brain Barrier

**DOI:** 10.3390/pharmaceutics13010038

**Published:** 2020-12-29

**Authors:** Gizem Rüya Topal, Mária Mészáros, Gergő Porkoláb, Anikó Szecskó, Tamás Ferenc Polgár, László Siklós, Mária A. Deli, Szilvia Veszelka, Asuman Bozkir

**Affiliations:** 1 Department of Pharmaceutical Technology, Faculty of Pharmacy, Ankara University, Yenimahalle, Ankara 06560, Turkey; gizemruya.topal@sbu.edu.tr; 2 Institute of Biophysics, Biological Research Centre, Temesvári krt. 62, H-6726 Szeged, Hungary; meszaros.maria@brc.hu (M.M.); porkolab.gergo@brc.hu (G.P.); szecskoaniko@gmail.com (A.S.); polgar.tamas@brc.hu (T.F.P.); siklos.laszlo@brc.hu (L.S.); deli.maria@brc.hu (M.A.D.)

**Keywords:** ApoE, blood–brain barrier, donepezil, drug delivery to brain, solid lipid nanoparticle, targeted drug delivery

## Abstract

Pharmacological treatment of central nervous system (CNS) disorders is difficult, because the blood–brain barrier (BBB) restricts the penetration of many drugs into the brain. To solve this unmet therapeutic need, nanosized drug carriers are the focus of research efforts to develop drug delivery systems for the CNS. For the successful delivery of nanoparticles (NPs) to the brain, targeting ligands on their surface is necessary. Our research aim was to design a nanoscale drug delivery system for a more efficient transfer of donepezil, an anticholinergic drug in the therapy of Alzheimer’s disease across the BBB. Rhodamine B-labeled solid lipid nanoparticles with donepezil cargo were prepared and targeted with apolipoprotein E (ApoE), a ligand of BBB receptors. Nanoparticles were characterized by measurement of size, polydispersity index, zeta potential, thermal analysis, Fourier-transform infrared spectroscopy, in vitro release, and stability. Cytotoxicity of nanoparticles were investigated by metabolic assay and impedance-based cell analysis. ApoE-targeting increased the uptake of lipid nanoparticles in cultured brain endothelial cells and neurons. Furthermore, the permeability of ApoE-targeted nanoparticles across a co-culture model of the BBB was also elevated. Our data indicate that ApoE, which binds BBB receptors, can potentially be exploited for successful CNS targeting of solid lipid nanoparticles.

## 1. Introduction

Alzheimer’s disease (AD) is a chronic neurodegenerative disease that ultimately leads to cognitive decline, memory impairment, aphasia, and behavioural issues. At present, around 50 million people are affected by AD worldwide, and this number is estimated to triple by 2050 [1]. Although therapeutic interventions for AD are intensively investigated, there is no approved drug formulation that is able to cure the disease [1]. There are five drugs approved by the Food and Drug Administration (FDA) for AD therapy, which provide modest benefit for cognitive symptoms [1]. These drugs do not stop or prevent the neurodegenerative process, only delay the progressive cognitive decline [1]. The main targets of modern therapeutics are biosynthetic pathway components of amyloid plaque or neurofibrillary tangle formation, as well as the regulation of inflammation in the central nervous system (CNS) [2]. Several small molecular drugs (such as secretase inhibitors) and biopharmacons (such as anti-amyloid β antibodies) were found to be effective in transgenic animal models of AD, yet a great number of clinical trials related to the therapy of AD have failed over the years due to several reasons [3]. An important aspect of successful therapies is that drugs reach their adequate location within the body and are able to interact with their cellular targets. In the case of AD and other neurodegenerative diseases, drug permeation from the blood to the brain parenchyma is hindered by the barrier systems of the CNS, including the blood–brain barrier (BBB) [3]. While the majority of drugs cannot cross the BBB due to their size, hydrophilic nature, or charge, brain capillary endothelial cells express a large number of blood-to-brain transport systems that can facilitate the entry of compounds to the brain [4,5]. Peptides and proteins necessary for proper neural function, such as insulin, transferrin, or lipoproteins, are transported across the BBB via receptor-mediated transcytosis [5]. Other nutrients, such as glucose, amino acids, or vitamins, enter the brain via carrier-mediated transport [4,6]. Several approved, commercially available neurotherapeutics are ligands of solute carriers (SLCs) and use the carrier-mediated pathway to enter brain endothelial cells [6]. For example, l-DOPA, a well-known drug in the treatment of Parkinson’s disease, is a ligand of LAT1/SLC7A5.

Donepezil is a widely used drug for the treatment of AD with mild-to-moderate symptoms since the 1990s. It acts as a reversible acetylcholinesterase inhibitor that increases the concentration of acetylcholine in cholinergic synapses. As cholinergic signaling plays in important role in the process of memory formation, elevated levels of acetylcholine in these synapses can alleviate the symptoms of dementia to some extent [1]. A major problem with donepezil, however, is that although it can cross the BBB [7], many unwanted side effects are reported to arise in the periphery including nausea, diarrhea, muscle spasms, hepatotoxicity, insomnia, and cardiac arrhythmia [1]. One strategy to increase brain penetration and reduce peripheral side effects of drugs is to encapsulate them into lipid- or lipid-polymer hybrid nanoparticles (NPs) [8,9]. These NPs are favorable, as they are biocompatible, biodegredable, and can accommodate both hydrophilic and hydrophobic drugs as cargo with controlled release properties [10]. Solid lipid nanoparticles (SLNs) consist of a core that is solid at body temperature and are stabilized by surfactants [11]. SLNs are especially promising drug delivery vehicles as they combine the versatility of vesicular NPs with the high stability of polymeric nanocarriers [12].

To ensure sufficient drug delivery to the brain, NPs need to be specifically targeted to bind physiological transport systems at the BBB [13]. Among the different strategies, exploitation of the receptor-mediated pathway in brain endothelial cells is the most widely investigated. In this approach, receptors of insulin, transferrin, or lipoproteins are targeted with their respective ligands, peptides, or antibodies on the surface NPs [5,14]. The binding of targeting molecules to brain endothelial receptors triggers internalization of receptor-NP complexes, which then results in transcytosis of a fraction of NPs across the BBB. Apolipoprotein E (ApoE) associates with lipids to form lipoproteins and plays a key role in the transport and uptake of cholesterol in the brain [15]. ApoE3, an isoform of ApoE, has high affinity to the low-density lipoprotein receptor (LDLR) and several low-density lipoprotein-associated receptors (LRPs). As these receptors are highly expressed by both brain endothelial cells and neurons, ApoE has become one of the main candidates as a targeting ligand of NPs for drug delivery to the brain [15,16]. Functionalization with ApoE has already been shown to elevate the BBB permeability of polymeric or albumin NPs [17,18,19] or SLNs [20,21,22], yet no previous studies investigated the effectiveness of ApoE-targeting for the delivery of donepezil in brain endothelial cells and neurons. Culture models of the BBB are important tools to test NPs and predict their brain penetration [23].

In this work, our aim was to develop an ApoE-targeted and SLN-based formulation of the anti-Alzheimer’s drug donepezil to increase its specific brain penetration. We determined that donepezil was efficiently encapsulated in SLNs with a favorable release profile. We then showed that ApoE-targeting of SLNs, carrying donepezil and the fluorescent dye rhodamine B as cargo, elevated the uptake of NPs in primary rat and human hCMEC/D3 brain endothelial cells, as well as human SH-SY5Y neuronal cells. Finally, we demonstrated that ApoE-targeting of donepezil-loaded SLNs increased the permeability of the cargo of NPs across a co-culture model of the BBB.

## 2. Materials and Methods

### 2.1. Materials and Reagents

All reagents were purchased from Sigma-Aldrich Kft. (Budapest, Hungary) (part of Merck Life Science, Darmstadt, Germany) unless otherwise indicated.

### 2.2. Cell Cultures

#### 2.2.1. Isolation and Culture of Primary Rat Brain Endothelial Cells, Pericytes, and Astroglias

For primary cultures of brain endothelial cells and pericytes, brains were obtained from three-week old, or for glial cell culture from two-day old, Wistar outbred rats (Harlan Laboratories, Bicester, UK) of both sexes. The animals were fed on standard rodent chow and water *ad libitum* and were kept under a 12 h light/dark cycle in the conventional animal house of the Biological Research Centre, Szeged. Organ harvest from animals was performed following the regulations of the 1998 XXVIII. Hungarian law and the EU Directive 2010/63/EU about animal protection and welfare.

Isolation of primary rat brain endothelial cells (RBECs), pericytes, and astrocytes were performed according to the method described in our previous studies [7,24,25]. After isolation, RBECs were seeded onto collagen type IV (100 μg/mL) and fibronectin (25 μg/mL) coated culture dishes (Corning Costar, New York, NY, USA) and were cultured in DMEM/HAM’s F-12 medium (Gibco, Waltham, MA, USA) supplemented with 15% plasma-derived bovine serum (PDS, First Link, Wolverhampton, UK), 10 mM HEPES, 100 μg/mL heparin, 5 μg/mL insulin, 5 μg/mL transferrin, 5 ng/mL sodium selenite (ITS, Pan‑Biotech, Aidenbach, Germany), 1 ng/mL basic fibroblast growth factor (bFGF, Roche, Basel, Switzerland), and 50 μg/mL gentamicin. During the first three days of culture, the medium of endothelial cells was completed with 3 μg/mL puromycin to eliminate P-glycoprotein negative, contaminating cell types [26]. After the first three days of culture, the amount of PDS was decreased from 15% to 10% in the culture medium.

Primary rat brain pericytes were isolated using the same method as for RBECs, except that pericytes were plated onto culture dishes (VWR International, Radnor, PA, USA) coated with collagen type IV (100 μg/mL). Primary cultures of rat glial cells were prepared from one-day-old Wistar rats, and cells were plated onto uncoated 75 cm^2^ flasks (TPP, Trasadingen, Switzerland). Both pericytes and astrocytes were cultured in DMEM medium (low glucose, Gibco, Waltham, MA, USA) supplemented with 10% fetal bovine serum (FBS, Pan-Biotech, Aidenbach, Germany) and 50 μg/mL gentamicin.

#### 2.2.2. Culture of Human Cerebral Microvascular Endothelial Cell Line hCMEC/D3

The hCMEC/D3 cell line (Merck Millipore Ltd., Budapest, Hungary) was grown in MCDB 131 medium (Pan Biotech) supplemented with 5% FBS, 1% GlutaMAX (Life Technologies, Carlsbad, CA, USA), 1% chemically defined lipid concentrate (Life Technologies, USA), 10 μg/mL ascorbic acid, 1.4 μM hydrocortisone, 100 μg/mL heparin, 1 ng/mL basic fibroblast growth factor, ITS, and 50 μg/mL gentamicin [27]. For uptake studies and 96 well plates for toxicity tests, hCMEC/D3 cells (passage number ≤35) were cultured in 24 well plates coated with rat-tail collagen (100 μg/mL). After reaching confluency, cells received 10 mM lithium chloride for 24 h before experiments to enhance BBB properties [7].

#### 2.2.3. Culture of Human Neuroblastoma Cell Line SH-SY5Y

SH-SY5Y cells (ATCC^®^ CRL-2266) were grown in DMEM/HAM’s F12 medium (Gibco, Waltham, MA, USA) supplemented with 10% FBS until reaching confluency. Cell differentiation was initiated by the addition of 10 μM retinoic acid dissolved in cell culture medium containing 0.5% dimethyl-sulfoxide and 2% FBS for five days [28]. For experiments, only differentiated cultures of SH-SY5Y were used.

### 2.3. Preparation of SLNs

Donepezil-loaded SLNs (DON-SLNs) were prepared by using a homogenization–sonication method [29], and the effects of various formulation parameters on the physicochemical properties of the nanoparticles were investigated. The formulation of DON-SLNs such as type of lipid and surfactant, their different ratios, the quantity of active substance, the time/speed of homogenization and sonication steps, and the type of aqueous solution was optimized based on previous results of the group [8,29] and studies from the literature [9]. Briefly, the lipid dynasan 116 (IOI Oleochemical, Hamburg, Germany) was heated to 75–80 °C and the active substance, donepezil HCl (Deva Holding A.Ş., İstanbul, Turkey), was dispersed in the lipid phase. Tween 80 was dissolved in phosphate buffered saline (PBS, pH 9.0) and heated to 75–80 °C. To label the particles, rhodamine B (RhB, 1 mg/100 mg lipid) was used and added to the formulations at the lipid phase step. The water phase was slowly added to the oil phase and mixed with the high-speed mixer (Ultra-Turrax T-25, IKA, Königswinter, Germany) at 9600 rpm for 5 min. Subsequently, the mixtures were sonicated (Sonopuls, Bandelin Gmbh, Berlin, Germany) for 2 min at 90% intensity, then let to cool to room temperature [29,30]. The formulations were finally centrifuged at 4000× *g* for 20 min at 4 °C three times using Amicon Ultra-4 centrifuge tubes (30K MKWCO, Merck, Darmstadt, Germany), and the pellets were collected and resuspended in serum and phenol red free culture medium.

### 2.4. Apolipoprotein E Functionalization of SLNs

For brain targeting, SLNs were modified with ApoE. The functionalization of nanoparticles with ApoE started by the biotinylation of ApoE (recombinant human ApoE3; Peprotech, London, OH, USA), followed by the addition of the functionally active avidins onto the surface of SLNs using DSPE-PEG-avidin [20]. For the biotinylation of ApoE, 0.5 mg ApoE and 3.75 mg biotin were dissolved in PBS at 4 °C and incubated for 4 h. To remove the free biotin, the biotinylated ApoE solution was dialysed by using 10K MWCO dialysis bag in PBS at 4 °C for 8 h, with four buffer changes. SLNs were prepared by adding 2 mg of DSPE-PEG-NH_2_ in the lipid phase as described before, so the surface of SLNs had amino terminal groups, which could be conjugated with the carboxyl group of avidin. The avidin solution was prepared in PBS at a concentration of 5 mg/mL, then 0.6 mg 1-ethyl-3-(3-dimethyl-aminopropyl)-carbodiimide (EDC) was added to this solution and stirred for 30 min at room temperature. The avidin solution was added to DSPE-containing SLNs and incubated for 2 h at room temperature. To remove the excess avidin, SLNs were dialysed in a 10K MWCO dialysis bag in PBS overnight at 37 °C. To bind the biotinylated ApoE to the avidin-conjugated SLNs, biotinylated ApoE solution, and avidin conjugated SLNs were stirred in equal volumes for 40 min and finally centrifuged at 4000× *g* for 20 min at 4 °C three times using Amicon Ultra-4 centrifuge tubes (30K MWCO, Merck, Darmstadt, Germany), and the pellets were collected (APOE-DON-SLNs) in serum and phenol red free culture medium.

### 2.5. Characterization of SLNs

#### 2.5.1. Particle Size, Polydispersity Index, and Zeta Potential

The size, polydispersity index (PDI) and zeta potential of SLNs were measured in three replicates using dynamic light scattering (Malvern Zetasizer Nano ZS, Malvern, UK). For the size and zeta potential measurements, SLN samples were diluted with ultrapure water at 200 µg/mL concentration. The suspension was then placed in the laser particle counter. All measurements were taken at 25 °C. Each sample was measured in triplicate.

#### 2.5.2. Determination of the Encapsulation Efficiency

For the calculation of the encapsulated donepezil HCl, an indirect method was used by measuring the donepezil HCl content of supernatants. SLN samples were centrifuged using Amicon Ultra-4 centrifuge tubes at 4000× *g*, and the active substance in the supernatant was detected by HPLC (Agilent 1260 Infinity, Agilent Tech., Waldbronn, Germany). The mobile phase was composed of methanol and water at a ratio of 85:15 at a flow rate of 0.5 mL/min. The injection volume was 20 μL and donepezil HCl was eluted at about 4.5 min and detected at 270 nm and 37 °C. The encapsulation efficiency (*EE*%) was calculated by using the following equation:

$$EE\;\left(\%\right)\;\mathrm{for}\;\mathrm{donepezil}\;\mathrm{HCl}=\left(\frac{\mathrm{Amount}\;\mathrm{of}\;\mathrm{total}\;\mathrm{drug}-\mathrm{Amount}\;\mathrm{of}\;\mathrm{loaded}\;\mathrm{drug}}{\mathrm{Amount}\;\mathrm{of}\;\mathrm{total}\;\mathrm{drug}}\right)\times 100$$

To determine the amount of encapsulated *RhB* in SLNs, the formulations were dissolved in 1% Triton X-100 in distilled water and measured by a spectrofluorometer (Fluorolog 3, Horiba Jobin Yvon, Edison, NJ, USA) at 559 nm excitation and 582 nm emission wavelengths. The concentrations were determined from a fluorescence calibration curve. The *EE* (%) was calculated by using the following equation:

$$EE\left(\%\right)\;\mathrm{for}\;RhB=\left(\frac{\mathrm{Amount}\;\mathrm{of}\;\mathrm{Rho}\;B\;\mathrm{in}\;\mathrm{the}\;\mathrm{NP}\;\mathrm{sample}}{\mathrm{Amount}\;\mathrm{of}\;\mathrm{Rho}\;B\;\mathrm{in}\;\mathrm{the}\;\mathrm{Triton}\;X\;\left(\%1\right)sol.}\right)\times 100$$

#### 2.5.3. Transmission Electron Microscopy of Nanoparticles

To characterize the morphology of DON-SLNs and APOE-DON-SLNs, the particles were observed by transmission electron microscopy (TEM; JEOL JEM-1400Flash, JEOL Ltd., Tokyo, Japan) operating at 120 kV. The samples were mounted on 150 mesh copper grids, stained with 10 μL 2% uranyl acetate in 50% ethanol for 3 min. After staining, samples were dried under a Petri dish for 2 h before the electron microscopic evaluation. The particles were recorded at 15,000–80,000× magnification with a 16 MP Matataki Flash scientific complementary metal–oxide–semiconductor (sCMOS) camera (JEOL).

#### 2.5.4. In Vitro Release Studies

The in vitro release of donepezil HCl from nanoparticles was detected using the dialysis membrane method. Donepezil HCl-loaded SLN samples (1 mL) were added to dialysis bags (12K MWCO) in dissolution medium (pH 7.4, PBS containing 0.1% Tween 80). The membrane was placed into 50 mL of dissolution medium and shaken horizontally in water bath (GFL 1083, GFL mbH, Burgwedel, Germany) at 50 rpm at 37 °C. At specific time points (0.25, 0.5, 1, 2, 4, 6, 8, 12 and 24 h), 1 mL of sample was taken, and 1 mL fresh dissolution medium was added. The samples were filtered by 0.45 nm syringe filters, and donepezil HCl concentrations were determined by using HPLC. All experiments were performed in triplicate for each of the samples.

#### 2.5.5. Thermal Analysis Using Differential Scanning Calorimetry

Thermal analysis of donepezil HCl, dynasan 116 lipid, DON-SLNs, and APOE-DON-SLNs were carried out using differential scanning calorimetry (DSC, Shimadzu DSC-60, Kyoto, Japan) to determine the physical state of the materials. About 2–3 mg of each sample was weighted, compressed into an aluminium pan, and analysed at a temperature range of 25 to 300 °C at a heating rate of 5 °C/min. All data acquired were processed on TA 60 universal analyser software and glass transition temperatures (Tg) were detected.

#### 2.5.6. Fourier-Transform Infrared Spectroscopy

Fourier-transform infrared (FTIR) spectrums of donepezil HCl, dynasan 116 lipid, DON-SLNs, and APOE-DON-SLNs were obtained by Shimadzu IRAffinity-1S spectrometer (Shimadzu, Kyoto, Japan). The spectra were recorded in the IR range from 650 to 4000 cm^−1^.

#### 2.5.7. Stability Studies

For stability studies, SLNs were kept at 4 °C in the dark for six months. Stability was examined with particle size, polydispersity index, and zeta potential measurements of SLNs at the beginning and end of the six-month period.

### 2.6. Cell Viability Assays

#### 2.6.1. Colorimetric Cytotoxicity Tests

The yellow 3-(4,5-dimethyltiazol-2-yl)-2,5-diphenyltetrazolium bromide (MTT; Sigma M5655, Budapest, Hungary) dye is taken up by cells and converted into blue formazan crystals by mitochondrial and cytoplasmic enzymes. Only living cells can convert MTT, so this test determines cell metabolic activity and viability. Decrease in dye reduction correlates to cell damage. SH-SY5Y cell cultures were treated with SLNs diluted in culture medium in the concentration range of 0.03–10 µg/mL for 2 h. MTT dye was prepared in phenol red-free medium at 0.5 mg/mL final concentration. After cell treatment, medium was removed and 0.5 mg/mL MTT solution was added to the cells for 4 h at 37 °C. Formazan crystals produced by living cells were dissolved in 100 µL/well dimethyl sulfoxide on a rotating shaker for 10 min. Absorbance was detected by a multiwell plate reader at 570 nm (Fluostar Optima, BMG Labtechnologies, Ortenberg, Germany). Cell viability was calculated as the percentage of dye reduction by culture medium-treated cells (control group).

#### 2.6.2. Impedance Measurement

Kinetics of RBEC and hCMEC/D3 cells responses to SLNs treatment were monitored by impedance measurement (RTCA-SP instrument; ACEA Biosciences, San Diego, CA, USA). Impedance measurement is label-free, real time, non-invasive, and correlates linearly with adherence and growth of cells. After background measurements, cells were seeded at a density of 6 × 10^3^ cells/well in collagen coated 96-well plates with integrated gold electrodes (E-plate 96, ACEA Biosciences, San Diego, CA, USA). Cells were cultured for 5–7 days in CO_2_ incubator at 37 °C. When the growth of cultures reached a plateau phase, cells were treated with SLNs (1 and 10 µg/mL) diluted in culture medium and monitored for 24 h. Cell index was defined as R_n_-R_b_ at each time point of measurement, where R_n_ is the cell-electrode impedance of the well when it contains cells, and R_b_ is the background impedance of the well with the medium alone. Cell index values reflect cell number and viability.

### 2.7. Cellular Uptake Studies

RBEC, hCMEC/D3, and SH-SY5Y cells were seeded in 24-well plates (Corning Costar, New York, NY, USA) at the concentration of 3 × 10^4^ cells/well. The confluent monolayers were incubated with 10 µg/mL DON-SLNs and APOE-DON-SLNs diluted in culture medium for 2 h at 37 °C in a CO_2_ incubator on a horizontal shaker (150 rpm). After incubation, cells were washed three times with ice cold PBS supplemented with 1% bovine serum albumin (BSA) and once with acid stripping buffer (glycine 50 mM, NaCl 100 mM, pH 3) to remove cell surface-associated SLNs. Finally, cells were lysed in PBS with 1% Triton X-100 detergent and the fluorescent signal was detected with a spectrofluorometer (Fluorolog 3, Horiba Jobin Yvon, Edison, NJ, USA) at 549 nm excitation and 572 nm emission wavelengths.

### 2.8. BBB Co-Culture Model and Permeability Assay

For the permeability studies we used a triple co-culture BBB model in which primary rat brain endothelial cells, pericytes, and astrocytes are cultured together in a transwell system [7,24]. Astroglial cells were passaged (8.5 × 10^4^ cells/cm^2^) to collagen type IV (100 μg/mL) coated 12-well plates (Corning Costar, New York, NY, USA). To prepare the co-culture model, pericytes (P2) were seeded (1.5 × 10^4^ cells/cm^2^) to the bottom side of tissue culture inserts (Transwell, polycarbonate membrane, 3 μm pore size, Corning Costar, USA) coated with collagen type IV (100 μg/mL). Brain endothelial cells were seeded (7.5 × 10^4^ cells/cm^2^) to the upper side of the culture insert membrane coated with Matrigel (growth factor reduced, Corning Costar, USA). Then the inserts containing brain endothelial cells and pericytes on the two sides of the membrane were placed to 12-well plates containing astrocytes at the bottom. Both the upper and lower fluid compartments of the model received endothelial cell culture medium supplemented with 550 nM hydrocortisone. The three cell types were cultured together for four days before permeability experiments.

The tightness of the BBB co-culture model was verified by measurement of transendothelial electric resistance (TEER) by an EVOM voltohm meter (World Precision Instruments, Sarasota, FL, USA) combined with STX-2 electrodes. TEER of coated, but cell-free, filters were subtracted from measured TEER values. When appropriate TEER values (210 ± 18 Ω × cm^2^) were obtained, the model was used for experiments. Cells were treated in the upper/donor compartment (0.5 mL) with DON-SLNs and APOE-DON-SLNs (10 μg/mL) diluted in phenol red free DMEM/F12 supplemented with 1% PDS and 1% ITS for 2 h. To test the function of our BBB model, the flux of permeability marker molecules sodium fluorescein (SF, 376 Da) and Evans blue-labeled serum albumin (EBA, 67 kDa) was determined across the endothelial monolayers. After treatments, samples were collected from the lower/acceptor compartments (1.5 mL) and measured with spectrofluorometer (Fluorolog 3) at 549 nm excitation and 572 nm emission wavelengths. The apparent permeability coefficients (P_app_) were calculated as described previously [31] by the following equation:

$$P_{\mathrm{app}}=\frac{\Delta {\left[C\right]}_{A}\times V_{A}}{A\times {\left[C\right]}_{D}\times \Delta t}$$

Briefly, P_app_ (cm/s) was calculated from the concentration difference of the cargo in the acceptor compartment (Δ[*C*]*_A_*) after 24 h. [*C*]_*D*_ is the concentration in the donor compartment at 0 h, *V_A_* is the volume of the acceptor compartment (1.5 mL), and A is the surface area available for permeability (1.12 cm^2^).

### 2.9. Statistical Analysis

Data are presented as means ± standard error of mean (SEM) or standard deviation (SD). Statistical analyses were performed using GraphPad Prism 8 software (Graphpad PRISM 5, Graphpad Software Inc., San Diego, CA, USA). Means were compared using unpaired *t* test or one‑way ANOVA followed by Dunnett’s posttest. Differences were considered statistically significant at *p* < 0.05. All experiments were repeated at least two times, and the number of parallel samples in each experiment was 4–8.

## 3. Results

### 3.1. Characterization of SLNs

#### 3.1.1. Size, Charge, Encapsulation Efficiency, and Morphology

**Figure 1:**
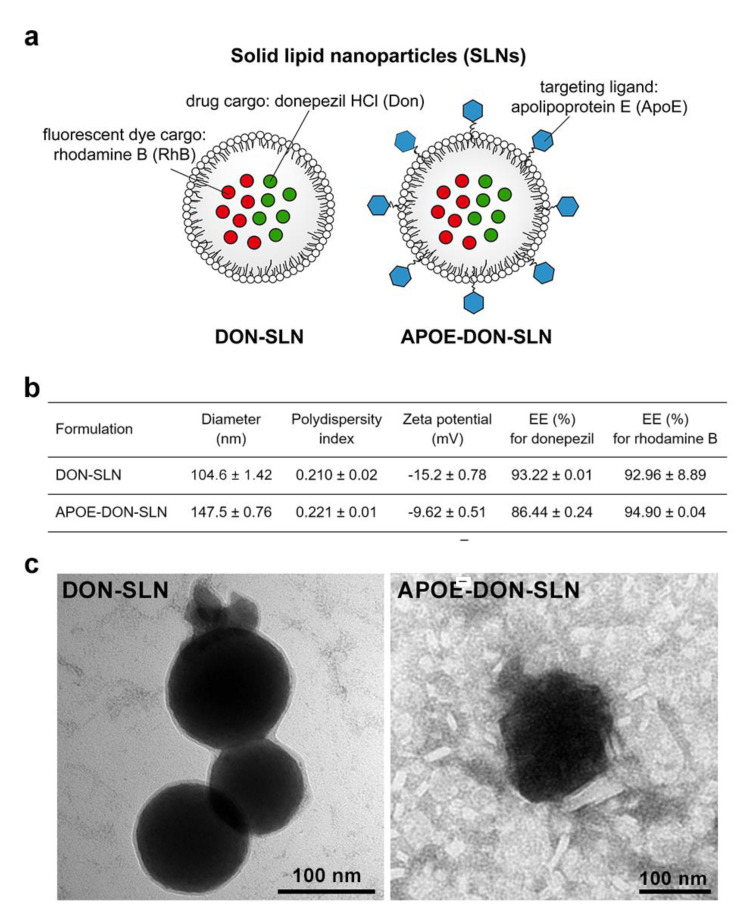
(**a**) Schematic drawing of non-targeted (DON-SLN) and ApoE targeted (APOE-DON-SLN) donepezil (DON) encapsulated solid lipid nanoparticles (SLN). (**b**) Main physico-chemical properties of SLNs. Values presented are means ± SD. EE%: encapsulation efficiency. (**c**) Transmission electron microscopy images of DON-SLNs and APOE-DON-SLNs. Scale bars: 100 nm for both DON-SLN and APOE-DON-SLN groups.

A schematic drawing of DON-SLNs and APOE-DON-SLNs is presented in Figure 1a. Figure 1b summarizes the main physico-chemical characteristics of the non-targeted and targeted groups. The mean diameter of the non-targeted nanoparticles was 104 nm, while the targeted SLNs were bigger (≥140 nm) due to the ApoE-functionalization. We measured a relatively narrow size distribution by dynamic light scattering indicated by PDI values ~0.2 in both groups (Figure 1b). The zeta potential of targeted particles was slightly negative, whereas non-targeted SLNs had a more negative surface charge. The encapsulation efficiencies of the cargo donepezil were 93% and 86% in DON-SLNs and APOE-DON-SLNs, respectively, while for RhB the EE% was between 92–94%, which was also very high. The morphology of the nanoparticles was observed by TEM (Figure 1c). The particles had mostly spherical shapes and their sizes were comparable with the results obtained from the Malvern Zetasizer measurements (Figure 1b).

#### 3.1.2. In Vitro Release Studies

**Figure 2:**
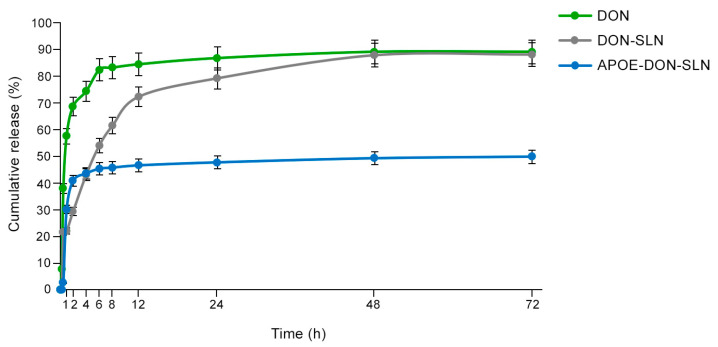
In Vitro donepezil HCl release profiles from donepezil aqueous solution (DON), non-targeted SLNs (DON-SLN), and SLNs functionalized with ApoE (APOE-DON-SLN).

The in vitro release of donepezil HCl from the targeted and non-targeted formulations were compared with donepezil HCl solution (Figure 2). The release profile of donepezil from DON-SLNs showed an initial burst release followed by slower release phase (Figure 2). In the first hour, 21% of the encapsulated drug was released from DON-SLNs compared to the 58% of the free drug solution. DON-SLNs showed sustained/prolonged maximum release of 89% at 72 h. Free drug solution reached 85% of the total amount of donepezil HCl release at 8 h, which was earlier than drug release from the nanoformulations. Comparing the release profile of DON-SLNs and APOE-DON-SLNs, the amount of donepezil HCl from particles at 72 h was 89% and 50%, respectively, idicating that ApoE targeted nanoparticles had a slower release profile.

#### 3.1.3. Thermal Analysis Using Differential Scanning Calorimetry (DSC)

**Figure 3:**
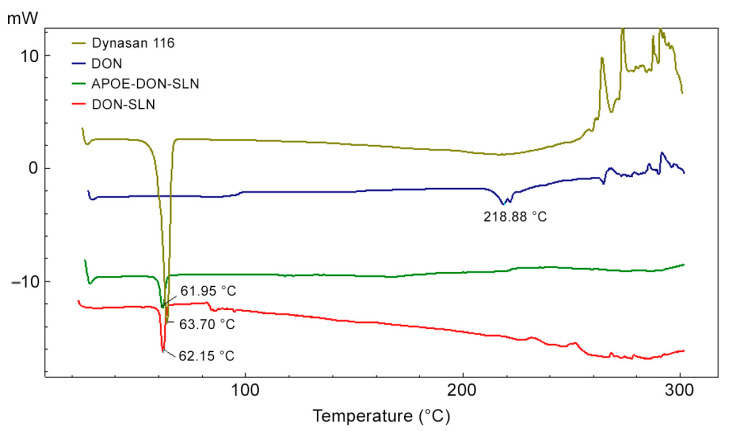
DSC thermograms of dynasan 116, donepezil HCl (DON), non-targeted (DON-SLN), and targeted SLNs (APOE-DON-SLN).

The DSC thermograms of dynasan 116, donepezil HCl, DON-SLNs, and APOE-DON-SLNs are presented in Figure 3.

Peaks from donepezil HCl at 218.88 °C and dynasan 116 at 63.70 °C corresponded to the melting point of donepezil HCl and dynasan 116 [32,33]. In the cases of the nanoparticle formulations, the donepezil HCl peak disappeared on the DSC thermogram, suggesting that donepezil HCl was amorphously charged to the formulation and homogeneously dispersed in the lipid.

#### 3.1.4. FTIR Analysis

**Figure 4:**
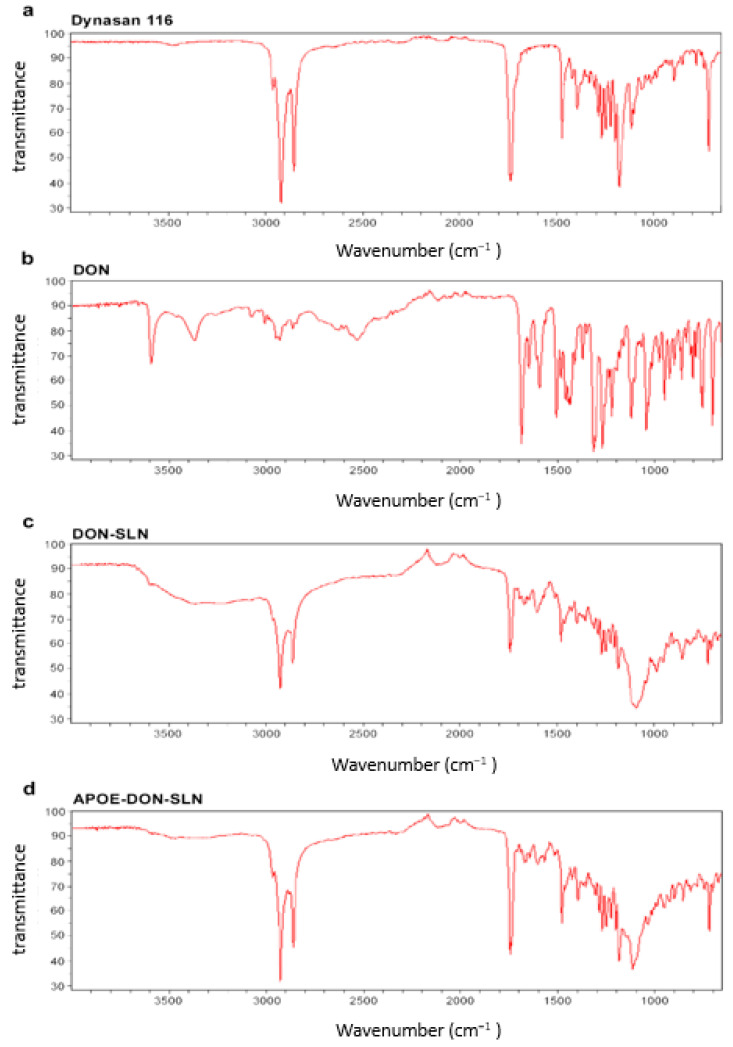
FTIR spectra of (**a**) dynasan 116, (**b**) donepezil HCl (DON), (**c**) non-targeted (DON-SLN), and (**d**) targeted SLNs (APOE-DON-SLN).

Possibilities of potential interaction between drug and other components used for preparation of SLNs were investigated by FTIR analyses (Figure 4).

These spectral data verified the chemical stability of donepezil HCl after the preparation of SLNs. The FTIR spectrum of donepezil HCl showed characteristic peaks at 3583, 3004, 2942, 1681 and 1589 cm^−1^ in accordance with literature data [32,34]. These peaks were not seen in the spectra of SLNs. The results confirmed that donepezil HCl was totally dispersed in lipid structures (Figure 4).

#### 3.1.5. Stability Studies

After six months of storage of SLNs at 4 °C, the particle size slightly but the polydispersity index significantly increased (Table 1). These results showed that the particles tended to aggregate over time, but size values were still below 200 nm. Zeta potential values also increased significantly at the end of the six-month period, from −15.2 to −18.9 in the case of non-targeted particles and −9.62 to −17.3 in ApoE-targeted SLNs (Table 1).

### 3.2. Effect of SLNs on Cell Viability

**Figure 5:**
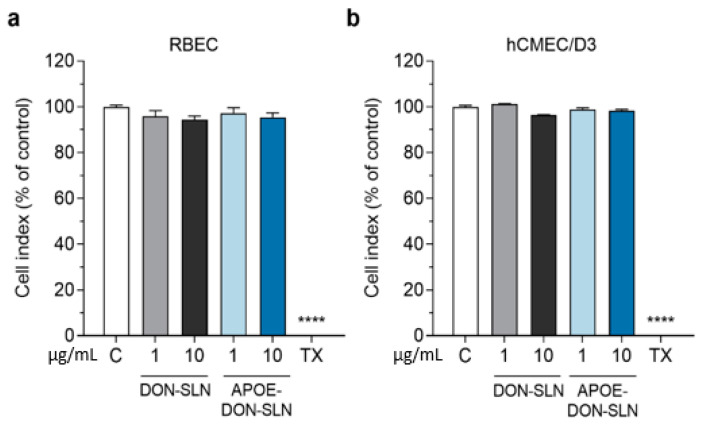
Effect of non-targeted (DON-SLN) and ApoE-targeted SLNs (APOE-DON-SLN) on the viability of (**a**) primary rat brain endothelial cells (RBEC) and (**b**) the human brain endothelial cell line hCMEC/D3 after 2 h of incubation, monitored by impedance measurement. Values presented are means ± SEM and are given as a percentage of control. Statistical analysis: one-way ANOVA followed by Dunnett’s posttest; **** *p* < 0.0001 compared to the control group; *n* = 6–8. C: culture medium-treated control group; TX: Triton X-100 treated cells, indicating maximal cellular toxicity.

To determine a safe treatment concentration of nanoparticles for further experiments, we monitored the response of brain endothelial cells to incubation with non-targeted or ApoE targeted SLNs containing donepezil in 1 and 10 µg/mL concentrations for 2 h (Figure 5).

After 2 h, neither DON-SLN nor APOE-DON-SLN treatments decreased the impedance of cell layers compared to the culture medium-treated control group, which indicated good cell viability (Figure 5). For further experiments we selected the 10 µg/mL concentration, which could be considered as a safe concentration.

**Figure 6:**
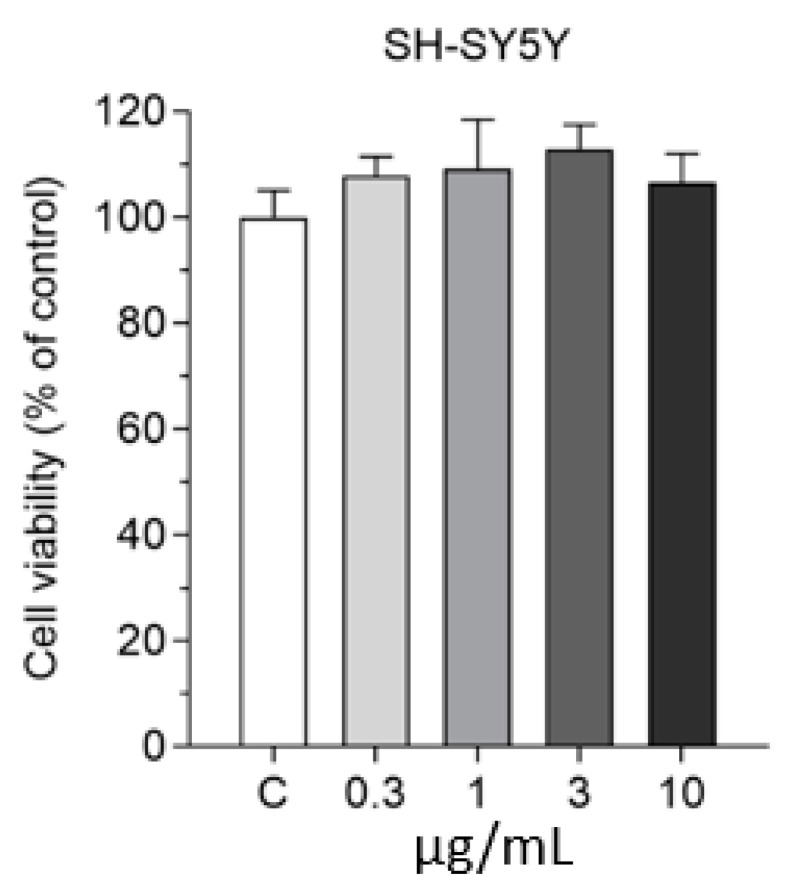
Effect of non-targeted SLNs (DON-SLN) on the viability of differentiated SH-SY5Y human neuronal cells measured by MTT test. Values presented are means ± SEM and are given as a percentage of control. Statistical analysis: one-way ANOVA followed by Dunnett’s posttest; *n* = 6–8. C: culture medium-treated control group.

We also tested the effect of SLNs on the viability of differentiated SH-SY5Y human neuronal cells in the 0.3–10 µg/mL concentration range for 2 h by MTT viability test. All tested concentrations were safe for neurons (Figure 6); therefore, based on these results, we selected the 10 µg/mL concentration for further uptake studies.

### 3.3. Cellular Uptake of SLN Cargo

**Figure 7:**
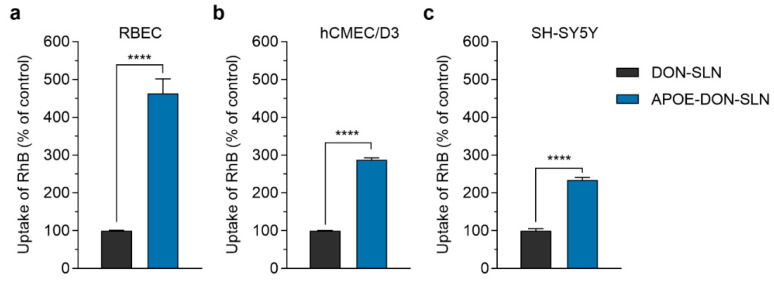
Cellular uptake of rhodamine B (RhB) cargo encapsulated in non-targeted (DON-SLN) and ApoE-targeted SLNs (APOE-DON-SLN) in (**a**) cultured primary rat brain endothelial cells (RBEC), (**b**) human brain endothelial cells (hCMEC/D3), and (**c**) in differentiated SH-SY5Y neuronal cells after 2 h of incubation. Values presented are means ± SEM. Statistical analysis: unpaired t test between DON-SLNs and APOE-DON-SLNs; **** *p* < 0.0001; *n* = 6.

The cellular uptake of DON-SLNs and APOE-DON-SLNs (10 µg/mL) were tested on rat and human BBB models and SH-SY5Y neuronal cells (Figure 7). In concordance with our hypothesis, the presence of ApoE targeting ligand on the surface of SLNs increased the uptake of RhB cargo in all tested cell types (Figure 7). In primary rat brain endothelial cells, the uptake of RhB-labeled donepezil cargo formulated in ApoE-targeted NPs was more than four times as high (463%) as cargo encapsulated in non-targeted particles after 2 h of incubation (Figure 7a).

Similarly to the rat model, in human brain endothelial cells, we also observed a significant difference in the uptake of cargo between non-targeted and targeted SLN-treated groups (APOE-DON-SLN: 288% compared to DON-SLN; Figure 7b). Compared to brain endothelial cells, we observed lower, but still two-fold, difference in the uptake of cargo between DON-SLNs and APOE-DON-SLNs in differentiated SH-SY5Y human neuronal cells (APOE-DON-SLN: 234% compared to DON-SLN; Figure 7c).

### 3.4. Permeability of SLN Cargo across the Blood-Brain Barrier Co-Culture Model

**Figure 8:**
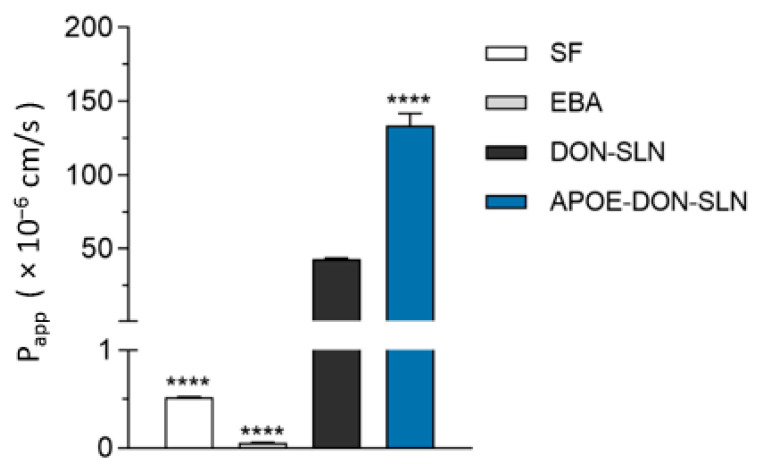
Permeability of RhB cargo encapsulated in non-targeted (DON-SLN) or ApoE-targeted SLNs (APOE-DON-SLN) across the BBB model (10 µg/mL concentration, 2 h). Values presented are means ± SEM. Statistical analysis: one-way ANOVA followed by Dunnett’s posttest, where **** *p* < 0.0001, compared to the DON-SLN group; *n* = 4. P_app_: apparent permeability coefficient, SF: sodium fluorescein (376 Da), EBA: Evans blue-labeled bovine serum albumin (67 kDa).

We tested the penetration of both DON-SLNs and APOE-DON-SLNs across the co-culture model of the BBB. To evaluate the integrity of our model, we also measured the penetration of the small paracellular permeability marker sodium fluorescein and the large biomolecule albumin across the BBB. The permeability of the model for both SF and EBA was very low (0.52 × 10^−6^ cm/s and 0.06 × 10^−6^ cm/s), reflecting a tight barrier (Figure 8). These P_app_ values for permeability marker molecules are in accordance with our previous results [7,25,35]. The penetrations of all tested RhB-labeled SLNs were several times higher than that of the marker molecules (Figure 8). Targeting the particles with ApoE resulted in 3.2-fold increase in the permeability of SLNs (P_app_ = 133.4 × 10^−6^ cm/s) compared to the penetration of non-targeted SLNs (P_app_ = 42.7 × 10^−6^ cm/s) across the BBB model (Figure 8).

## 4. Discussion

As the global population continues to age, the prevalence of neurodegenerative diseases, like AD, also increases [36]. The lack of suitable and effective treatments generated intense research efforts to understand the cytological, genetic, and molecular aspects of the disease. AD patients have an impairment of the cholinergic–neurotransmitter systems due to the suppression of acetylcholinesterase (AChE) activity and activation of the glutamatergic system plays a significant role in the pathology [37]. Based on these data, the four FDA approved drugs in AD therapy are the NMDA inhibitor memantine; AChE inhibitors donepezil, rivastigmine, and galantamine; and the fifth medication is the combination of donepezil with memantine [1]. Unfortunately, these drugs are not curative and only ameliorate the symptoms and increase the quality of life by improving the cognitive and motor functions [1]. Conventional, *per os* applied medicines, including powder, capsule, tablet, or liquid formulations, have limitations such as low bioavailability, rapid first pass metabolism, poor pharmacokinetics, and high dose requirement, which may result in significant side effects in non-target organs [10,12]. NPs, especially targeted ones, can improve the bioavailability and kinetic profile of CNS drugs in biological systems and help to safely deliver drugs to specific sites of action in the brain [38].

### 4.1. Lipid Nanoparticles for Brain Delivery

In the last 25 years, lipid NPs have become one of the most important drug delivery systems studied for the treatment of brain disease [39]. These lipid nanocarriers are more biocompatible compared to polymeric or inorganic nanoparticles, and they have an inherent ability due to their small size and lipid nature, to better penetrate the BBB [39]. SLNs can be easily functionalized with targeting molecules like peptides or antibodies compared to polymer-based nanostructures, where in certain cases complex chemical reactions are needed for the functionalization. The other disadvantage of polymeric nanocarriers can be that the therapeutic cargo is more likely released in bursts and less in a sustained form. SLNs show a controlled release of various drugs including antioxidants, enzymes, or other therapeutic agents for long periods of time [39].

Several types of targeted SLNs, regarding the cargo and targeting molecules, have been reported for potential AD treatment. The cargo can be a therapeutic molecule inhibiting Aβ aggregation or promoting degradation of amyloid plaques. Another group of agents for encapsulation in SLNs are natural compounds like curcumin, resveratrol, or piperine due to their neuroprotective characteristics. Classical AD drug galantamine was also formulated in lipid NPs [40], but we found no studies focusing on donepezil.

Regarding functionalization of SLNs for brain targeting, we also found diverse approaches. SLNs and liposomes functionalized with the Aβ 1–42 ligands phosphatidic acid and cardiolipin effectively targeted Aβ, while the plain SLNs could not [41]. An anti-transferrin receptor monoclonal antibody, OX26 mAb, was used as a targeting ligand for SLNs containing resveratrol and grape extracts that was tested on a human BBB co-culture model [42]. In these experiments the uptake of the OX26-functionalized SLNs was more efficient than that of the non-targeted SLNs in human brain-like endothelial cells. The same OX26 antibody as well as its combination with anti-Aβ antibody DE2B4 could increase the uptake of polymeric NPs loaded with iAβ5 peptide in porcine brain capillary endothelial cells, a culture model of the BBB [43].

### 4.2. ApoE-Targeting of SLNs for Crossing the BBB

The expression of LDLR and LRP family members is high at the BBB, and these receptors are also present on neurons [15,16,44]. They bind different types of apolipoproteins, and among them, ApoA-I and B-100 have been considered as targeting molecules for NPs to transport drugs across the BBB [45]. Humans have three ApoE isoforms, ApoE2, ApoE3, and ApoE4, from which ApoE4 show a strong genotype effect on the risk of sporadic and late-onset forms of AD [46]. Among the isoforms, ApoE3, which is five times more common in the population than ApoE4, has a strong binding affinty to the LDLR and LRPs presented on the surface of brain endothelial cells and neurons [44]. Two recent studies have demonstrated that NPs functionalized with ApoE targeting ligands are potential effective brain delivery systems. SLNs targeted with an ApoE peptide crossed hCMEC/D3 monolayers, a simplified human in vitro model of the BBB, and also increased brain penetration in mice [22]. Another group also found that ApoE functionalization elevated the uptake and permeability of SLNs in the hCMEC/D3 model [21]. However, SLNs in these studies did not contain therapeutic cargo.

In our present experiments, we developed and characterized SLNs containing donepezil as active therapeutic agent that were targeted with ApoE ligand. Physico-chemical properties of lipid NPs influence biodistribution: SLN formulations with a particle size of about 200 nm can stay longer in the blood flow, may lengthen the contact time with the BBB, and increase brain penetration of drugs [38]. Because of these reasons, we aimed to prepare SLN formulations with a particle size below 200 nm and high encapsulation efficiency. Regarding surface charge, as another important parameter, NPs with neutral or slightly negative surface charge showed higher brain penetration in comparison with positively charged untargeted NPs [47]. Negatively charged lipid NPs are generally less toxic and have higher stability compared to positively charged NPs [48]. The SLNs prepared in our study were negatively charged due to the characteristic properties of dynasan 116. The zeta potential values of our SLNs were between −9 and −18 mV (Figure 1 and Table 1), and we measured a more negative charge in the case of non-targeted NPs compared to the targeted ones. The therapeutic effects of NPs are dependent on both physicochemical properties and specific targeting. Surface charge is one of the important factors that determine the cellular interaction of NPs with barriers [38]. In general, cationic nanoparticles are supposed to be better internalized than neutral and negatively charged NPs, but recent comparative studies prove that neutral or negatively charged NPs are more efficient for drug delivery across the blood–brain barrier [49]. In our previous studies, we successfully used negatively charged NPs functionalized with ligands of brain endothelial transporters for targeting the blood–brain barrier [15,35,50]. The data of the six-month stability experiments also support that the zeta potential of our formulations does not affect significantly the size or aggregation of the SLNs.

Encapsulation efficiency of donepezil HCl was 93% for non-targated SLNs and 86% for targeted SLNs. We hypothesize that functionalization may lead to the loss of the drug present on or near the surface of SLNs as it was demonstrated in another study, in which also a decrease in the encapsulation efficiency was measured for lactoferrin targeted SLNs [51]. We measured a slower drug release profile in APOE-DON-SLNs. The DSPE-PEG linker that we used for the preparation of ApoE targeted formulations might cause a stabilization of donepezil HCL in the lipid matrix of SLN because of its interactions with the lipid. In addition, binding of new groups on the surface of SLNs can induce a barrier for degredation reactions for lipid structure of NPs [20] that may also slow the release of encapsulated donepezil from APOE-DON-SLNs. PEG is well known to stabilize lipid nanoparticles by forming a hydrated polymeric layer on the surface of lipid NPs [52]. We hypothesize that the PEG-APOE functionalization also formed such a hydrated layer on the surface of SLNs, which was responsible for the observed slower and lower dissolution of donepezil. We detected a short rapid release and a long steady release stage for APOE-DON-SLNs. A similar slow and low drug release was obtained from lipid nanoparticles during in vitro release experiments, while enhanced brain penetration was found in vivo [53]. In our experiments APOE-DON-SLNs penetrated to BBB model more than DON-SLNs despite the slower release profile indicating that targeting may play a more decisive role in barrier crossing.

In our experiments, we found significantly elevated uptake of the targeted APOE-DON-SLNs not only in rat and human brain endothelial cells, but also in a human differentiated neuronal cell line, as compared to the non-targeted NPs. Furthermore, we measured a two-fold higher penetration of ApoE-targeted rhodamine labeled NPs across a co-culture model of the BBB vs. DON-SLNs. The main physicochemical characteristics of different rhodamine dyes are close to each other [54]. In our recent study, the P_app_ of rhodamine 123 was 7 × 10^−6^ cm/s [55] on the same rat BBB model that we used in the present work. Based on the literature data combined with this result, we suppose that the permeability of rhodamine on our culture BBB model is below 10 × 10^−6^ cm/s, which is far smaller than the penetration of the rhodamine labeled SLNs in the present study. Our findings are in accordance with data from the literature, such as ApoE-targeting being efficient to increase uptake or penetration of SLNs, but in these studies SLNs were prepared without drug cargo and tested only on brain endothelial cell monocultures [21,22]. Our data extend these observations with the demonstration that ApoE as a targeting ligand could enhance both the uptake and permeabilty of SLNs loaded with the AD drug donepezil using BBB and neuron culture models.

The limitation of our study is that the penetration of ApoE targeted lipid nanoparticles were tested on a rat BBB model system in culture inserts. In further studies, testing the permeability of APOE-DON-SLNs on a human BBB model and investigation of the mechanism of the cellular uptake of the nanoparticles would be important. Novel microfluidic devices [56,57] would allow in future investigations the use of a dynamic flow condition in which brain endothelial cells can be exposed to fluid flow and shear stress, which may influence the uptake and transfer of NPs.

## 5. Conclusions

One of the key problems for the treatment of neurological diseases is to deliver an effective amount of therapeutics into the brain without significant side effects in the periphery. NPs and their proper functionalization are in the focus of research efforts to develop successful drug delivery systems for AD therapy. Nanocarriers can accumulate in several peripherial organs, and only specific BBB-targeting can increase the ratio of NPs penetrating the CNS. To ensure this relative brain specificity, it is important to functionalize the NPs with targeting molecules which are ligands of physiological transporters of the BBB and able to trigger active and specific transport mechanisms across the BBB. In the present study, we show that donepezil-loaded and rhodamine labeled SLNs targeted with ApoE ligand increased the uptake of these lipid nanoparticles in both cultured human brain endothelial cells and neurons. Furthermore, the permeability of these ApoE-targeted and donepezil loaded nanoparticles was also elevated across a co-culture model of the BBB. Our data indicate that ApoE can not only help solid lipid nanoparticles loaded with donepezil to cross the BBB but also facilitate their entry to neurons, which may be beneficial for CNS targeting of therapeutics.

## Figures and Tables

**Table 1:** Particle size, polydispersity index, and zeta potential values of non-targeted (DON-SLN) and ApoE- targeted SLNs (APOE-DON-SLN) determined at the day of preparation and at the end of a six-month period. Values presented are means ± SD (*n* = 3).

Day	Formulation	Diameter (nm)	Polydispersity Index	Zeta Potential (mV)
0	DON-SLN	104.6 ± 1.4	0.21 ± 0.02	−15.2 ± 0.8
180		108.2 ± 0.5	0.28 ± 0.01	−18.9 ± 1.1
0	APOE-DON-SLN	147.5 ± 0.8	0.22 ± 0.01	−9.6 ± 0.5
180		151.0 ± 3.5	0.41 ± 0.02	−17.3 ± 0.2

## Data Availability

All data are available upon request.
